# Prevalence and factors associated with e-cigarette use among aspiring media professionals: A cross-sectional survey at a vocational college

**DOI:** 10.18332/tid/235831

**Published:** 2026-09-22

**Authors:** Congcong Li, Mingbiao Zhang, Jing Zhao, Anliang Zhao, Jiangtao Ye, Yusui Zhao, Xiaofeng Hou

**Affiliations:** 1 Department of Critical Care Medicine, Sir Run Run Shaw Hospital, School of Medicine, Zhejiang University, Hangzhou, China; 2 Dongyang Center for Disease Control and Prevention, Dongyang, Jinhua, China; 3 Hengdian College of Film and Television, Dongyang, Jinhua, China; 4 Zhejiang Provincial Center for Disease Control and Prevention, Hangzhou, China

**Keywords:** e-cigarette use, college students, vocational education, tobacco control, China

## Abstract

**INTRODUCTION:**

E-cigarette use among college students is an emerging public health concern, particularly among those in media-related fields who may influence youth as future role models. This study examined the prevalence and factors associated with e-cigarette use among students at a vocational performing arts college in eastern China.

**METHODS:**

A cross-sectional census survey was conducted from April to May 2025 at Hengdian College of Film and Television. All enrolled students present on campus were invited to complete an online questionnaire adapted from the National Tobacco Survey for College Students. Data on demographic characteristics, e-cigarette use, exposure to advertising and health information, and knowledge of tobacco hazards were collected. Chi-squared tests and binary logistic regression were used to identify factors associated with current e-cigarette use.

**RESULTS:**

Among 9197 valid respondents (99.70% response rate; 69.90% female; mean age 18.83 years), the current e-cigarette use rate was 2.05%. Multivariate analysis identified the following significant associated factors: inadequate knowledge versus adequate knowledge (adjusted odds ratio, AOR=4.69; 95% CI: 3.44–6.40), maternal smoking versus neither parent smokes (AOR=9.44; 95% CI: 3.46–25.76), junior year versus freshman (AOR=3.46; 95% CI: 1.96–6.11), both parents smoking vs neither (AOR=3.04; 95% CI: 1.71–5.42), witnessing public smoking versus not (AOR=2.67; 95% CI: 1.82–3.91), male versus female (AOR=2.36; 95% CI: 1.74–3.20), sophomore year versus freshman (AOR=1.44; 95 CI: 1.09–1.91), and having smoking roommates versus not (AOR=1.26; 95% CI: 1.07–1.48). Nearly half reported exposure to e-cigarette advertising, while over 60% encountered anti-e-cigarette information.

**CONCLUSIONS:**

The associations identified highlight the need for further longitudinal investigation into modifiable factors and environmental influences. Special attention should be given to students with maternal smoking and those in smoking-normalized environments.

## INTRODUCTION

Tobacco use remains a serious global public health concern, causing more than 8 million deaths worldwide each year. In China, over 300 million smokers consume 45% of the world’s cigarettes, with tobacco-related deaths already exceeding one million annually^1^. In 2003, Chinese engineers invented the e-cigarette; since 2007, these devices have become increasingly prevalent in high-income countries, often marketed as smoking cessation aids in some nations^2,3^. In recent years, e-cigarettes have gradually gained popularity in China^4^. As e-cigarettes become more accessible and manufacturers continue to expand their marketing and promotion efforts, the trajectory of e-cigarette use appears to have deviated from the original harm reduction intent^5^. However, evidence suggests that e-cigarettes may not only fail to facilitate successful smoking cessation but may also lead to e-cigarette dependence and pose substantial health risks^6,7^.

College students are at a critical developmental stage, particularly susceptible to health-risk behaviors as they transition to independent living in unfamiliar environments^8^. While China’s ‘100% smoke-free campus policy’ has reduced traditional tobacco use^9^, e-cigarettes have become increasingly prevalent on campuses due to their fashionable image^10^. Recent national estimates indicate a current e-cigarette use rate of approximately 1.7% among Chinese college students^11^, showing an upward trend annually^12^. In China, substantial variations exist in e-cigarette use among college students across different regions and types of institutions. Data from multiple cities reveal distinct geographical disparities in usage rates among this population^13^. Differences are also observed by institution type: a 2021 national survey in China demonstrated that e-cigarette use rates were highest in vocational colleges, followed by provincial and local institutions, and lowest in centrally-administered or provisionally-ministerial co-constructed universities^10^.

Current research indicates that e-cigarette use among college students is shaped by both individual and environmental factors^14^. Individual determinants include demographic characteristics such as gender, age, and academic year. Knowledge level also plays a key role—students with limited awareness of the risks associated with e-cigarettes are more likely to use them^15^. Environmental factors are equally influential: social circles, including friends’ and roommates’ smoking behaviors, along with parental smoking (particularly maternal smoking), are strongly associated with student use^16,17^. In addition, exposure to e-cigarette advertising across various media and retail settings increases the perceived social acceptability of smoking and strengthens intentions to try both e-cigarettes and conventional cigarettes^18^. Since the implementation of China’s 100% smoke-free campus policy, studies have shown that while many students recognize some health risks of e-cigarettes, their understanding remains fragmented. In particular, the perception that e-cigarettes are less harmful than traditional cigarettes persists^19^.

Hengdian College of Film and Television, established in 2006, is the only higher vocational institution in Zhejiang Province specializing in film and television education, located within the world’s largest film and television shooting base. Graduates pursue high-profile careers as screenwriters, directors, and performers, becoming public figures whose behaviors may influence youth who view them as role models. Research on tobacco use in this specific population remains scarce^20^, particularly regarding e-cigarette use. This study aimed to: 1) investigate the prevalence and factors associated with e-cigarette use among media-focused vocational college students; 2) assess students’ exposure to e-cigarette advertising and anti-e-cigarette health information; and 3) identify their preferred channels for receiving tobacco control knowledge.

## METHODS

### Study design and setting

This cross-sectional study was conducted from April to May 2025 at Hengdian College of Film and Television, a performing arts vocational institution in Zhejiang Province, eastern China. A census method was employed to survey all enrolled students present on campus during the survey period.

### Sampling and participants

The target population comprised all full-time students across all grades and majors enrolled as of December 2024. At the time of the survey, the college had a total of 13470 enrolled students, including 4789 freshmen, 4139 sophomores, and 4542 juniors. As the majority of juniors were engaged in off-campus internships, they were not included in this on-campus survey. Ultimately, 9223 students present on campus were invited to participate, with 9197 valid questionnaires collected, yielding a valid response rate of 99.70%.

### Data collection and measures

*Training of survey personnel*

Prior to the survey, all faculty members from participating departments and institutes underwent standardized training conducted by researchers from the Dongyang Center for Disease Control and Prevention. The training program comprised collective lectures, platform demonstrations, and simulation exercises based on pre-survey pilot tests. A unified technical manual was used throughout the training. The training covered the background and purpose of this tobacco survey, interpretation of core questionnaire items, and operational protocols for the Wenjuanxing platform^21^, ensuring that all personnel involved accurately understood the survey requirements, proficiently mastered the workflow, and could conduct the survey according to standardized protocols.

*Data collection process*

All survey data were uniformly collected using the Wenjuanxing online platform (https://www.wjx.cn), a professional survey tool widely utilized for data collection in academic research, enabling fully electronic documentation and real-time synchronization throughout the process. Prior to the survey launch, a designated researcher edited and pretested the questionnaire on the platform. After verifying the logical consistency of the questions and confirming the proper functionality of the completion and submission procedures, a unique QR code and weblink were generated for the survey.

Upon official launch, the survey access point was distributed through official departmental WeChat groups and other institutional communication channels. All eligible students were instructed to complete the questionnaire within the designated timeframe. To prevent duplicate submissions, the Wenjuanxing platform was configured to restrict responses based on IP address and WeChat ID verification, ensuring each participant could submit only once.

*Quality control measures*

Throughout the data collection phase, a dedicated researcher continuously monitored response progress via the Wenjuanxing administrative back-end and compiled daily participation statistics by department. Indicators such as completion time and missing item rates were reviewed in real time. Questionnaires completed in an unusually short time (below the reasonable response duration established during pretesting) or containing missing responses to key items were flagged accordingly.

To ensure data authenticity, the platform automatically captured and stored response data in real time, eliminating the need for manual data entry and minimizing the risk of transcription errors. Following data collection, all data underwent systematic cleaning. Invalid questionnaires, including those with extensive missing responses, logical inconsistencies, or clear indications of arbitrary answering (e.g. patterned responses or completion times below the minimum threshold), were excluded. A standardized survey dataset was subsequently established and subjected to data validity verification by specialists at the Dongyang Center for Disease Control and Prevention. Only datasets that passed this verification process were deemed eligible for subsequent analysis.

### Measurement

The questionnaire used in this survey was developed by the Dongyang Center for Disease Control and Prevention. It was adapted from the nationally standardized National Tobacco Survey for College Students^11^, with revisions made to reflect the specific characteristics of performing arts institutions. Following optimization through a pilot survey, the final version was titled the ‘Online Survey on Smoking Behaviors Among Students at Hengdian College of Film and Television’.

The questionnaire covered the following domains: respondents’ demographic information, e-cigarette use, sources of e-cigarettes and exposure to e-cigarette advertising, as well as knowledge regarding the health hazards of tobacco use. This study employed the 11-item Tobacco Hazard Knowledge Scale (total score: 16), developed in 2025 and demonstrating sound reliability and validity^22^. The scale includes six single-choice items and five multiple-choice items. Correct answers to multiple-choice questions were awarded 2 points, while single-choice items received 1 point each, for a total possible score of 16. Each of the 11 items achieved a content validity index (CVI) ranging from 0.621 to 0.728, and the overall Cronbach’s α coefficient was 0.710. The Kaiser-Meyer-Olkin (KMO) measure was 0.821, and Bartlett’s test of sphericity was significant (p<0.001), indicating adequate reliability and validity. Based on receiver operating characteristic (ROC) analysis using current e-cigarette use as the classification state variable, a cutoff score of ≥4 was adopted to identify participants with adequate knowledge, yielding a sensitivity of 80.8% and a specificity of 53.8%.

*Definition of key variables*

‘Preferred information channels’ were assessed by asking participants to select from a predefined list of eight channels (traditional mass media, government/official sources, online/social media platforms, formal education/training, healthcare services, academic sources, peer/community support, and non-profit organization activities) through which they would like to receive tobacco control knowledge.

*Exposure to e-cigarette advertising*

Was measured by asking whether participants had seen advertisements in any of nine specified locations (e.g. retail stores, supermarkets, online social media, television, etc.) in the past 30 days.

*Exposure to anti-e-cigarette health information*

Was similarly assessed across seven locations (internet/social media, television/radio, newspapers/magazines, retail outlets, etc.).

### Variables

Data were collected on the following variables: sex, age (years), ethnicity (Han vs minority), academic year (freshman, sophomore, junior), college (School of Film and Television Performance, School of Film and Television Production, School of Film and Tourism, School of Film and Economics, School of Film and Fine Arts, College of Continuing Education), parental smoking status (neither, both, father only, mother only, unknown/unclear), roommate smoking status (non-smoking, smoking, unknown/unclear), witnessed smoking in public during the past 7 days (no, yes), and knowledge level (inadequate vs adequate based on the cutoff score of ≥4).

### Statistical analysis

Data were analyzed using SPSS (Version 27.0). Descriptive statistics are presented as frequencies and percentages for categorical variables and means and standard deviations for continuous variables. The chi-squared test was employed to examine differences in the prevalence of current e-cigarette use across sociodemographic characteristics and other categorical variables in univariate analysis. Crude odds ratios (ORs) with 95% confidence intervals (CIs) were calculated for pairwise comparisons.

Binary logistic regression analysis was performed to identify factors independently associated with current e-cigarette use (the outcome variable). All variables that showed significant associations in univariate analyses (p<0.05) were entered simultaneously into the multivariate model as covariates to adjust for potential confounding. Adjusted odds ratios (AORs) with 95% CIs were calculated to estimate the strength of associations between each factor and current e-cigarette use after adjusting for potential confounders. All statistical tests were two-tailed, and a p<0.05 was considered statistically significant. Missing data were minimal (<1% of items) and were handled using pairwise deletion; no imputation was performed.

### Ethics

The study protocol was approved by the Ethics Committee of Dongyang Center for Disease Control and Prevention. The survey was anonymous, and all procedures were conducted with strict confidentiality. Informed consent was obtained from all participants prior to survey administration. All methods were performed in accordance with the relevant guidelines and regulations.

## RESULTS

### Participant characteristics

A total of 9197 valid questionnaires were analyzed (99.70% valid response rate). The mean age of participants was 18.83 years (SD=0.83). The majority were female (69.90%, n=6429). Freshmen comprised 58.66% (n=5395) of the sample, sophomores 38.24% (n=3517), and juniors 3.10% (n=285). The distribution across colleges ranged from 1.60% (College of Continuing Education, n=147) to 21.83% (School of Film and Tourism, n=2008). Detailed sociodemographic characteristics are presented in Table 1.

**Table 1:** Sociodemographic characteristics of the study participants, a cross-sectional survey, Hengdian College of Film and Television, Zhejiang, China, 2025 (N=9197)

*Characteristics*	*Category*	*n (%)*
**Sex**	Male	2768 (30.10)
	Female	6429 (69.90)
**Age** (years), mean (SD)		18.83 (0.83)
**Ethnicity**	Han	8941 (97.22)
	Minority	256 (2.78)
**Academic year**	Freshman	5395 (58.66)
	Sophomore	3517 (38.24)
	Junior	285 (3.10)
**College**	School of Film and Television Performance	1952 (21.22)
	School of Film and Television Production	1710 (18.59)
	School of Film and Tourism	2008 (21.83)
	School of Film and Economics	1582 (17.20)
	School of Film and Fine Arts	1798 (19.55)
	College of Continuing Education	147 (1.60)

### Prevalence of e-cigarette use and univariate associations

The overall current e-cigarette use rate was 2.05% (190/9197). In univariate analysis, significantly higher prevalence was observed among males (4.05%, OR=3.47; 95% CI: 2.58–4.66) compared with females (1.21%). Prevalence increased with academic year: freshmen 1.41%, sophomores 2.47% (OR vs freshmen=1.77; 95% CI: 1.32–2.38), and juniors 6.32% (OR vs freshmen=4.66; 95% CI: 2.73–7.96) (p for trend <0.001).

Parental smoking was strongly associated with e-cigarette use. Compared with students whose neither parent smoked (2.10%), e-cigarette use was significantly higher among those with maternal smoking only (8.93%, OR=4.55; 95% CI: 1.72–12.02), both parents smoking (7.14%, OR=3.58; 95% CI: 2.06–6.23), and those with unknown parental status (4.36%, OR=2.12; 95% CI: 1.14–3.95). Paternal-only smoking (1.56%) was not significantly different from the reference (OR=0.74; 95% CI: 0.54–1.02).

Environmental exposures also showed significant univariate associations. Students with smoking roommates had a higher prevalence (3.64%) compared with those with non-smoking roommates (1.53%, OR=2.40; 95% CI: 1.72–3.36). Witnessing public smoking in the past 7 days was associated with a much higher prevalence (20.38% vs 1.76%, OR=14.31; 95% CI: 10.57–19.37). Students with inadequate knowledge had substantially higher e-cigarette use (5.93%) compared with those with adequate knowledge (0.99%, OR=6.25; 95% CI: 4.26–9.18). All these associations were statistically significant (p<0.001). Results of univariate analyses are shown in Table 2.

**Table 2:** Association between sociodemographic factors and current e-cigarette use among Vocational Film and Media College students, a cross-sectional survey, Hengdian College of Film and Television, Zhejiang, China, 2025 (N=9197)

*Characteristics*	*Category*	*Total* *n*	*Current e-cigarette use* *n (%)*	*χ^2^/Z*	*p*
**Sex**	Male	2768	112 (4.05)	76.756	<0.001
	Female	6429	78 (1.21)		
**Academic year**	Freshman	5395	76 (1.41)	27.785	<0.001
	Sophomore	3517	68 (1.93)		
	Junior	285	18 (6.32)		
**Ethnicity**	Han	8941	183 (2.05)	0.582	0.446
	Ethnic Minority	256	7 (2.73)		
**College**	School of Film and Television Performance	1952	55 (2.82)	18.147	<0.001
	School of Film and Television Production	1710	29 (1.70)		
	School of Film and Tourism	2008	53 (2.64)		
	School of Film and Economics	1582	22 (1.39)		
	School of Film and Fine Arts	1798	26 (1.45)		
	College of Continuing Education	147	5 (3.40)		
**Parental smoking**	Neither	3993	84 (2.10)	55.298	<0.001
	Both	224	16 (7.14)		
	Father only	4626	72 (1.56)		
	Mother only	56	5 (8.93)		
	Unknown/unclear	298	13 (4.36)		
**Roommate smoking status**	Non-smoking	6251	97 (1.53)	30.335	<0.001
	Smoking	954	36 (3.64)		
	Unknown/unclear	1802	57 (3.07)		
**Witnessed smoking in public** (past 7 days)	No	8613	152 (1.76)	60.781	<0.001
	Yes	584	119 (20.38)		
**Knowledge level**	Inadequate	2006	119 (5.93)	189.557	<0.001
	Adequate	7191	71 (0.99)		

### Multivariate analysis

After adjusting for all variables that were significant in univariate analyses (sex, academic year, parental smoking, roommate smoking, witnessed public smoking, and knowledge level), several factors remained independently associated with current ecigarette use. Inadequate knowledge was strongly associated with increased odds of use compared with adequate knowledge (AOR=4.69; 95% CI: 3.44–6.40). Maternal smoking showed the largest effect, with students whose mother only smoked having nearly 9.5fold higher odds than those with neither parent smoking (AOR=9.44; 95% CI: 3.46–25.76), followed by both parents smoking (AOR=3.04; 95% CI: 1.71–5.42). Academic year also contributed significantly: juniors had more than three times the odds of freshmen (AOR=3.46; 95% CI: 1.96–6.11), and sophomores showed a moderate increase (AOR=1.44; 95% CI: 1.09–1.91). Witnessing public smoking in the past 7 days nearly tripled the odds (AOR=2.67; 95% CI: 1.82–3.91), and male sex was associated with more than double the odds compared with female (AOR=2.36; 95% CI: 1.74–3.20). Having smoking roommates had a modest but significant association (AOR=1.26; 95% CI: 1.07–1.48). In contrast, paternalonly smoking was not independently associated with ecigarette use (AOR=1.28; 95% CI: 0.92–1.77, p=0.138). Full results of the multivariate logistic regression are presented in Table 3.

**Table 3:** Binary logistic regression analysis of factors associated with current e-cigarette use, a cross-sectional survey, Hengdian College of Film and Television, Zhejiang, China, 2025 (N=9197)

*Variables*	*Category (comparison vs reference)*	*β*	*SE*	*Wald*	*p*	*AOR (95% CI)*
**Gender**	Male vs Female	0.857	0.156	30.245	<0.001	2.36 (1.74–3.20)
**Parental smoking**	Both smoke vs Neither smokes	1.113	0.295	14.269	<0.001	3.04 (1.71–5.42)
	Father only smokes vs Neither smokes	0.245	0.165	2.203	0.138	1.28 (0.92–1.77)
	Mother only smokes vs Neither smokes	2.245	0.512	19.245	<0.001	9.44 (3.46–25.76)
**Smoking roommates**	Present vs Absent	0.230	0.084	7.559	0.006	1.26 (1.07–1.48)
**Witnessed smoking in public** (past 7 days)	Yes vs No	0.981	0.196	25.126	<0.001	2.67 (1.82–3.91)
**Knowledge level**	Inadequate vs Adequate	1.545	0.158	95.518	<0.001	4.69 (3.44–6.40)
**Grade**	Sophomore vs Freshman	0.367	0.143	6.571	0.01	1.44 (1.09–1.91)
	Junior vs Freshman	1.240	0.291	18.195	<0.001	3.46 (1.96–6.11)
**Constant**	-4.638	0.276	281.823	<0.001		0.01

SE: standard error. AOR: adjusted odds ratio. CI: confidence interval. The model included all variables that were significant in univariate analyses: sex, academic year, parental smoking, roommate smoking, witnessed public smoking, and knowledge level. Adjusted odds ratios were obtained from a single binary logistic regression model.

### Advertising and health information exposure

Exposure to e-cigarette advertising in the past 30 days was reported by nearly half of participants (48.64%). The most common locations were e-cigarette experience stores or retail outlets (40.71%), shops/supermarkets/convenience stores (32.00%), and online social media (16.03%). Exposure to anti-e-cigarette health information was reported by 61.96% of participants, most commonly via the internet/social media (45.10%), television/radio (36.85%), and newspapers/magazines (24.16%). Detailed exposure patterns are presented in Table 4 (Panels A and B).

**Table 4:** Exposure to e-cigarette advertising, anti-e-cigarette health information, and preferred channels for tobacco control knowledge, a cross-sectional survey, Hengdian College of Film and Television, Zhejiang, China, 2025 (N=9197)

*Panel A: Exposure to e-cigarette advertising in the past 30 days*	
**Location of advertisement exposure**	**Respondents who saw advertisements n (%)**
E-cigarette experience stores or retail outlets	3744 (40.71)
Shops, supermarkets, convenience stores, or grocery stores	2943 (32.00)
Online social media (e.g. WeChat, QQ, Weibo)	1474 (16.03)
Television	1329 (14.45)
Websites (e.g. Taobao, JD.com)	1270 (13.81)
Newspapers or magazines	1134 (12.33)
Outdoor billboards	997 (10.84)
Radio	868 (9.44)
Social activities (e.g. sporting events, cultural/art performances)	523 (5.69)
Overall (saw advertisement in at least one location)	4473 (48.64)
***Panel B: Exposure to anti-e-cigarette health information in the past 30 days***	
**Location of exposure**	Respondents who saw the information n (%)
Internet (including social media)	4148 (45.10)
Television or radio	3389 (36.85)
Newspapers or magazines	2222 (24.16)
E-cigarette experience stores or retail outlets	2164 (23.53)
Shops, supermarkets, convenience stores, or grocery stores	2105 (22.89)
Outdoor billboards	1465 (15.93)
Social activities (e.g. sporting events, cultural/art performances)	949 (10.32)
Overall (saw information in at least one location)	5697 (61.96)
***Panel C: Preferred channels for receiving tobacco control knowledge***	
**Preferred channel**	Respondents who preferred this channel n (%)
Traditional and Mass Media (television, radio, newspapers, billboards, posters, etc.)	5839 (63.49)
Government or Official Sources (government health department websites)	5024 (54.63)
Online/Social Media Platforms (e.g. WeChat, QQ, Weibo)	4294 (46.69)
Formal Education/Training (e.g. lectures, class meetings)	3882 (42.21)
Healthcare Services (medical services and school doctor consultation)	3775 (41.05)
Academic Sources (magazines/academic journals)	3665 (39.85)
Peer/Community Support (recommendations from friends/family, quit-smoking groups, school clubs)	2949 (32.06)
Non-Profit Organization Activities (e.g. Red Cross campaigns)	2567 (27.91)

### Preferred information channels

Students’ preferred channels for receiving tobacco control knowledge were: traditional/mass media (63.49%), government or official sources (54.63%), online/social media platforms (46.69%), and formal education/training (42.21%). Healthcare services, academic sources, peer/community support, and non-profit organization activities were preferred by 41.05%, 39.85%, 32.06%, and 27.91% of respondents, respectively. Full results are shown in Table 4 (Panel C).

## DISCUSSION

This cross-sectional study found a current e-cigarette use rate of 2.05% among vocational performing arts students, with significant associations with inadequate knowledge, maternal smoking, upper year status, and environmental exposures. The observed prevalence is comparable to recent estimates from broader Chinese college student populations^11^ and provincial surveillance data from Zhejiang^23^, while slightly higher than the most recent surveillance data from Shanghai^24^. This subtle elevation is concerning given that these aspiring media professionals will occupy influential public roles where their behaviors may shape social norms, especially among youth.

Among individual-level modifiable factors, inadequate knowledge showed the strongest association with e-cigarette use, consistent with research demonstrating that knowledge deficits increase susceptibility to novel tobacco products^25,26^. This finding is particularly salient given that the perception of e-cigarettes as less harmful than traditional cigarettes persists among Chinese college students^26^, despite emerging evidence of substantial health risks including cardiovascular and respiratory diseases^7,8^. The relatively low cutoff score for adequate knowledge (≥4 out of 16) suggests that absolute knowledge levels may be suboptimal, highlighting the need for more comprehensive educational efforts^27^.

Maternal smoking exhibited the strongest association, far exceeding paternal-only smoking, which was not significant. This aligns with research suggesting maternal health behaviors exert particularly powerful influences on offspring, possibly due to closer emotional bonds and greater modeling effects in Chinese family contexts^28,29^. Both parents smoking further supports the importance of family environment, with implications for family-based interventions targeting parents, especially mothers.

Environmental exposures were consistently significant. Witnessing public smoking nearly tripled use odds, while smoking roommates showed a modest but significant association, underscoring the normative influence of visible smoking behaviors^26,27^. The high advertising exposure, particularly in retail settings, is concerning given evidence that such exposure increases smoking acceptability and intentions to try e-cigarettes^30,31^.

Encouragingly, over 60% of students reported exposure to anti-e-cigarette health information, predominantly through internet/social media and television/radio. This relatively high reach suggests that current health communication strategies are achieving moderate penetration, though there remains substantial room for improvement. Students’ preferences for receiving tobacco control knowledge through traditional mass media, government sources, and online platforms provide clear guidance for optimizing future health communication campaigns. The strong preference for official and mass media channels over peer support or non-profit organization activities suggests that students perceive authoritative sources as more credible, aligning with previous research on health information preferences among Chinese youth^32^. Notably, formal education and training were preferred by over 40% of respondents, indicating that school-based interventions remain a valuable and acceptable platform for reaching this population^33^.

The higher prevalence among males and upper year students reflects patterns consistently observed in tobacco research^34^, potentially attributable to gender differences in risk-taking behaviors, peer influences, and cumulative exposure to social environments where smoking is normalized. The particularly elevated association among juniors, despite their small proportion in the sample, may reflect the unique circumstances of students preparing to transition from campus to professional environments, a period when health behaviors may be particularly susceptible to change^35,36^.

### Limitations

Several limitations should be considered when interpreting these findings. First, the cross-sectional design precludes causal inferences; associations identified between factors and e-cigarette use should be interpreted as correlational rather than causal. Second, the study was conducted at a single vocational college specializing in performing arts, which may limit generalizability to other types of higher education institutions or student populations in different regions. Third, data were self-reported and may be subject to social desirability bias, potentially leading to underreporting of e-cigarette use, although anonymous survey administration likely mitigated this concern. Self-reported data may also introduce misclassification bias, particularly if participants under-report or over-report their e-cigarette use. Fourth, the knowledge scale, while demonstrating acceptable reliability and validity, yielded relatively modest sensitivity (80.8%) and specificity (53.8%) at the established cutoff, suggesting some misclassification may have occurred. Fifth, due to the timing of data collection (April–May 2025), a substantial proportion of junior students were engaged in off-campus internships and were not included, potentially introducing selection bias and limiting representativeness for upper year students. Although we adjusted for multiple covariates, residual confounding from unmeasured variables (e.g. peer influence outside the campus, personal attitudes) cannot be ruled out. Finally, the study did not assess dual use of traditional cigarettes and e-cigarettes, patterns of use frequency, or motivations for use, which represent important directions for future research.

## CONCLUSIONS

This study provides an examination of e-cigarette use among students at a vocational performing arts college in China, a population that will soon occupy influential public roles as media professionals. While the current e-cigarette use prevalence is relatively modest, the strong associations with modifiable factors – particularly inadequate knowledge, environmental exposures, and family smoking behaviors – highlight clear opportunities for further investigation. The pervasive exposure to e-cigarette advertising in retail settings, coupled with students’ preferences for receiving tobacco control information through mass media and official sources, suggests that future research should evaluate the effectiveness of communication strategies tailored to this population. Special attention should be directed toward students with maternal smoking, upper year students, and those in social environments where smoking is normalized. As these future media professionals will shape public perceptions and social norms through their work and public visibility, investing in their tobacco-related health literacy and preventing e-cigarette uptake carries implications that extend far beyond individual health outcomes to population-level tobacco control efforts. Longitudinal research is needed to track trajectories of e-cigarette use as these students transition into professional careers and to provide robust evidence for future prevention strategies.

## Data Availability

The data supporting this research are available from the authors on reasonable request.
